# Dinosaurian Soft Tissues Interpreted as Bacterial Biofilms

**DOI:** 10.1371/journal.pone.0002808

**Published:** 2008-07-30

**Authors:** Thomas G. Kaye, Gary Gaugler, Zbigniew Sawlowicz

**Affiliations:** 1 Department of Paleontology, Burke Museum of Natural History, Seattle, Washington, United States of America; 2 Microtechnics Inc., Granite Bay, California, United States of America; 3 Department of Geology, Jagiellonian University, Krakow, Poland; Paleontological Institute, Russian Federation

## Abstract

A scanning electron microscope survey was initiated to determine if the previously reported findings of “dinosaurian soft tissues” could be identified in situ within the bones. The results obtained allowed a reinterpretation of the formation and preservation of several types of these “tissues” and their content. Mineralized and non-mineralized coatings were found extensively in the porous trabecular bone of a variety of dinosaur and mammal species across time. They represent bacterial biofilms common throughout nature. Biofilms form endocasts and once dissolved out of the bone, mimic real blood vessels and osteocytes. Bridged trails observed in biofilms indicate that a previously viscous film was populated with swimming bacteria. Carbon dating of the film points to its relatively modern origin. A comparison of infrared spectra of modern biofilms with modern collagen and fossil bone coatings suggests that modern biofilms share a closer molecular make-up than modern collagen to the coatings from fossil bones. Blood cell size iron-oxygen spheres found in the vessels were identified as an oxidized form of formerly pyritic framboids. Our observations appeal to a more conservative explanation for the structures found preserved in fossil bone.

## Introduction

The previous discovery of soft, pliable tissues recovered from the dissolved remains of *Tyrannosaur* bone in 2005 [1], potentially marked a major turning point in the science of paleontology given that it extended the known range of preserved biomolecules by many orders of magnitude. The implication that these were preserved dinosaurian soft tissues held the promise of biologic investigations of extinct animals. The original discovery centered on several tyrannosaur specimens. From this single report, it could not be determined if this was a wholly unique one-of-a-kind preservation, or these structures remained undiscovered in other fossil material. Subsequent investigations [2] showed that these microstructures existed across a range of time and taxa and ruled out a one-time exceptional preservation. The previous work required that the fossil bone be dissolved in acid to expose the preserved microstructures.

A new line of investigation was undertaken to detect the material in unaltered bone. Furthermore these structures should be occurring commonly in bone from the same formations. This work expands on these initial investigations by examining the interior of the fossil dinosaur bone prior to dissolution in acid using scanning electron microscopy (SEM) and energy dispersive spectroscopy (EDS). The survey totaled more than 200 hours of SEM time, covered seven geologic formations and more than fifteen genera outlined in Table 1. The data and findings presented identify a bacterial biofilm that mimics soft structures previously thought to be biological tissue. This explanation is in marked contrast to the concept of an exceptional preservation scenario of dinosaurian soft tissue and represents a plausible alternative hypothesis.

**Table 1:** Specimens examined in survey.

Age	Formation	Genera
Pleistocene	Indet	*Mastodon*
Eocene	Chadron	*Brontothere*
		*Stylemys*
Paleocene	Wind River	Microsite indet.
Cretaceous	Hell Creek	*Edmontosaurus*
		*Triceratops*
		*Ankylosaurus*
		*Theropod* Indet
		*Champtosaurus*
		*Lepisosteus*
		*Aspideretoides*
	Lance	*Edmontosaurus*
		*Triceratops*
		*Ankylosaurus*
		*Theropod* Indet.
	Bearpaw Shale	*Mosasaurus*
		*Didymoceras*
	Pierre Shale	*Hoploscaphites*

Four categories of tissues were initially discovered in 2005 [1]: (A) Clusters of spheres that showed an iron-oxygen elemental signature appeared red under the light microscope. (B) Soft, branching, tube-like structures that contained spheres. (C) Free floating “osteocytes” complete with fillapodia and (D) a filamentous mass that remained pliable and elastic. Subsequent tests using immunochemistry showed positive for proteins [3]. Three of these structures were found commonly in this survey and discussed below.

## Materials and Methods

Scanning electron microscopes used were JEOL T300, Zeiss Supra and Cambridge S200. Light microscopes were Zeiss Axiomat, Nikon SMZ-U and Unitron. EDS spectra were taken with Kevex Delta 5, EDAX Apollo 40 running under EDAX Genesis/Pegasus and Link LZ5 running under WinEDS.

SEM specimens were prepared by pressure fracturing and selected pieces approximately 10 mm square were fixed to aluminum stubs with high purity carbon tabs. Initial specimens were uncoated to minimize potential disruption of internal contents. Subsequent specimens were gold coated with a Bio-Rad E5000 sputter coater or Denton Desk IV turbo coater with Pd or Pt target. Low dot-pitch element maps were run at 15 kV. The maps of individual elements were combined with a high-resolution secondary electron images to produce a high resolution colored element maps.

Modern biofilms were grown with the following method. Two gallons of water obtained from a local pond was placed in a new five gallon bucket and recirculated with a small pump at room temperature. Five new microscope slides were placed in the bottom with 100 mg of glucose nutrient added to the water. Water samples were taken every two days to monitor microbe population. One slide was removed and examined under the light microscope every few days to monitor biofilm accumulation rate. Slides were allowed to desiccate at room temperature over several days. Microbial communities could clearly be seen in the hydrated biofilms under the light microscope but subsequent examination under SEM showed only a smooth undulating profile.

Fourier Transform Infrared Spectroscopy (FT-IR) was used to investigate the specimens' molecular structure. A turtle carapace from the Hell Creek formation was selected for spectroscopy because of its proportionally large chambers in the trabecular bone that allowed scraping the coatings loose. Two milligrams of material was ground with 450 milligrams of potassium bromide (KBr) and pressed into a pellet using 8 tons pressure. Modern biofilms grown on microscope slides in pond water were allowed to desiccate for 7 days and 2.5 milligrams were pressed into a KBr pellet as above. A 2.5 milligram sample of desiccated tendon from a chicken was ground with KBr and pelletized. Spectrums were taken on a Nicolet 510P bench at 1 cm^−1^ resolution with a minimum of 15 scans. Infrared flux was matched within 5% for all specimens and a clean KBr pellet used for background subtraction between specimens. Excel cross correlation routines were used to determine percentage of similarity for spectrums.

**Figure 1:**
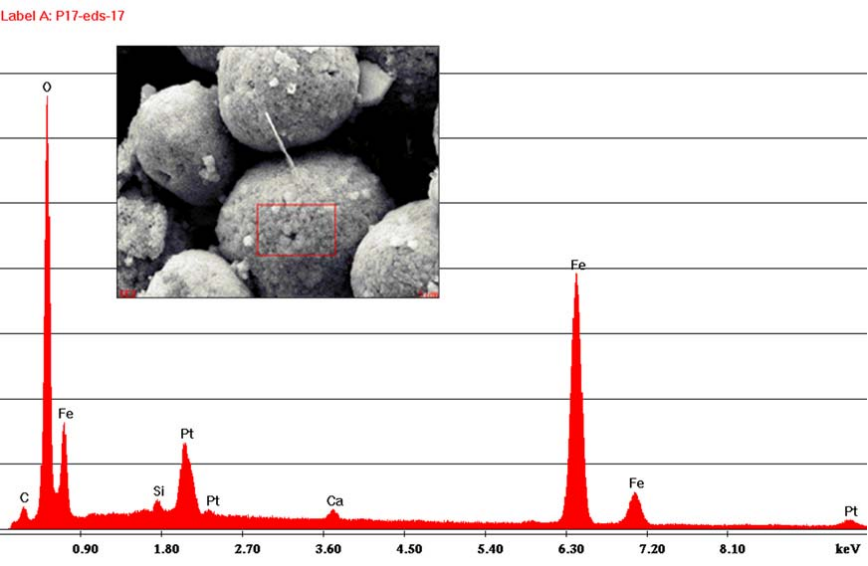
EDS spectrum of framboid. EDS spectrum of framboid showing an iron-oxygen signature. Pt is from coating for SEM. Area in red box was scanned for elements.

Framboids were individually extracted from fractured dinosaur bone fragments with a magnet and transferred to a carbon sticky tab on an SEM stub. EDS was performed on a small section of the sphere at 20 kV in the area shown with the spectrum in figure 1.

Multiple specimens were pressure fractured and 10–20 mm fragments selected for demineralization in 0.5 M ethylenediaminetetraacetic acid (EDTA) (pH 8.0) in individual plastic containers at room temperature. Resident times ranged from several days to several weeks depending on specimen resistance. Baths were changed at approximately three day intervals with fresh acid. Remaining structures were either photographed directly in the baths at low magnification 7–75× or removed for higher power imaging.

All specimens for carbon dating were handled under a flow hood with clean sterile gloves and instruments. The specimens were pressure fractured to reveal fresh surfaces. A bone fragment from the Lance formation was microscopically examined and coatings that appeared to have been dislodged were removed for analysis. Fifty milligrams of material were sent to Geochron Labs, Cambridge Mass. for accelerator mass spectrometry (AMS) analysis. The results were 139.01%±0.65 of modern (1950) of ^14^C activity.

## Results

**Figure 2:**
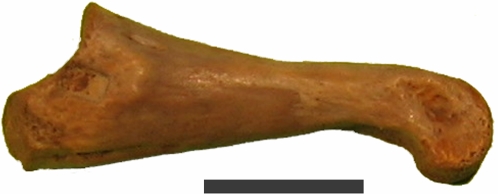
Well preserved complete bone used in initial investigation. Exceptionally well preserved small phalange from the Lance formation used for initial survey. No cracks or deformities present. Specimen was pressure fractured and directly examined under the SEM. UWBM 89327 Scale bar, 10 mm.

**Figure 3:**
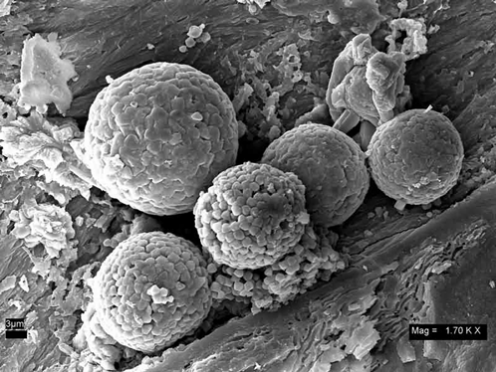
Iron oxide framboids. An iron oxide framboid cluster in dinosaur trabecular bone found commonly throughout time and taxa. At approximately 10 microns in diameter they are closely matched in size to red blood cells and typical pyrite framboids. UWBM 89327 Scale bar, 3 µm.

**Figure 4:**
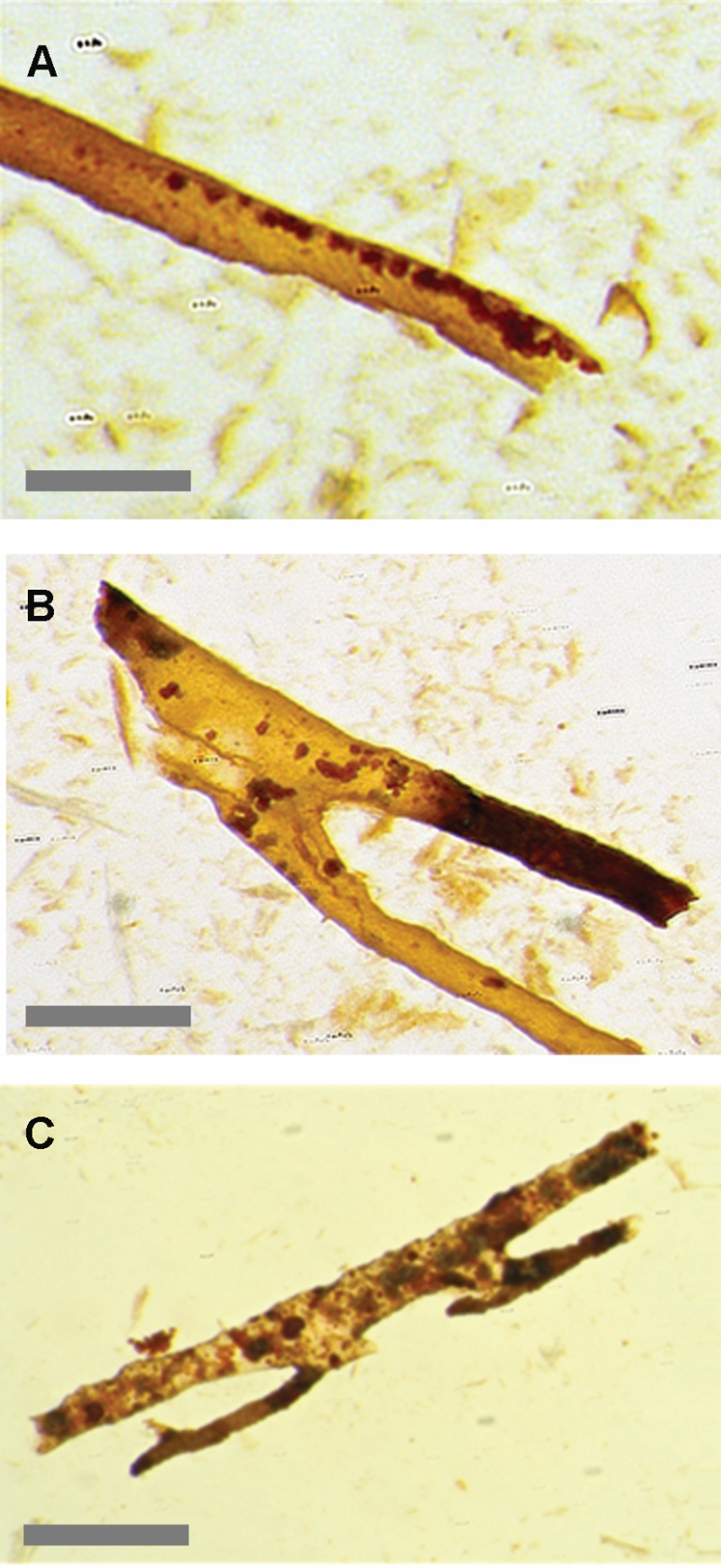
Tubular branching structures. Branching, transparent tube-like structures that match the porosity of the trabecular bone. Note small red grains that were found to be iron oxide framboids. These structures remain in acid baths after demineralization. Some are pliable, others frangible. Scale bars, 100 µm. Photos Z stacked, 7 images, unsharp mask, gamma adjusted.

The hollow morphology of the tyrannosaur femur supported the general idea that an exceptionally well-preserved bone may act as a containment vessel for biomolecules. To test this concept, a perfectly preserved turtle phalange showing no cracks or deformities (Fig. 2) was first selected for SEM examination. The specimen was pressure fractured and directly examined uncoated in the SEM. A cluster of spheres approximately 10 microns in diameter, similar to Fig. 3, was discovered almost immediately. Subsequent EDS showed an iron-oxygen signature. Continuing SEM surveys of multiple specimens from the Lance, Hell Creek, Chadron and Pierre Shale formations all showed similar iron-oxygen spheres ranging in size from 5–29 microns. Examination of demineralized specimens under the light microscope displayed small red spheres clustered in the tubular structures (Fig. 4A). Discovery of these spheres in an ammonite suture indicated they had no relationship to iron derived from blood. These spheres were identified as framboids which are seen world wide in black smokers, algal mats and are commonly found in sediments [4].

The second structure category consisted of soft, pliable, branching tubules with morphology closely resembling blood vessels. To further investigate this hypothesis, specimens from the Lance and Hell Creek formations were subjected to EDTA baths in order to determine if similar soft structures were present after dissolution of the bone. After demineralization, tubular branching structures that conformed to the size and shapes of the open vascular canals in the spongy bone were found remaining in the baths (Fig. 4 A–C). Some of these structures were pliable while others were frangible, consistent with previous discoveries [2]. Extensive EDS mapping was done to identify these coatings in the voids of the unaltered fossil bone.

**Figure 5:**
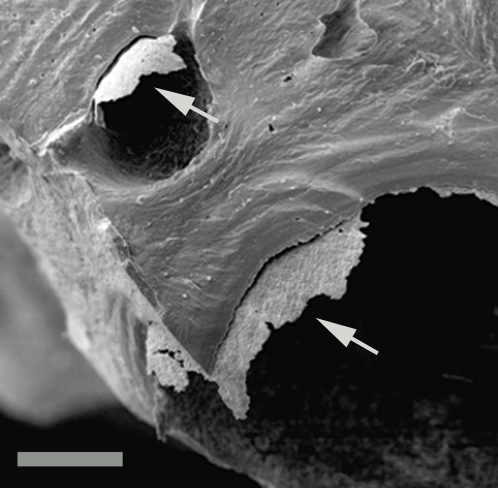
Coatings on vascular canal walls. SEM image of fractured bone showing coatings naturally peeling from vascular canal walls. UWBM 89324 Scale bar, 150 µm.

**Figure 6:**
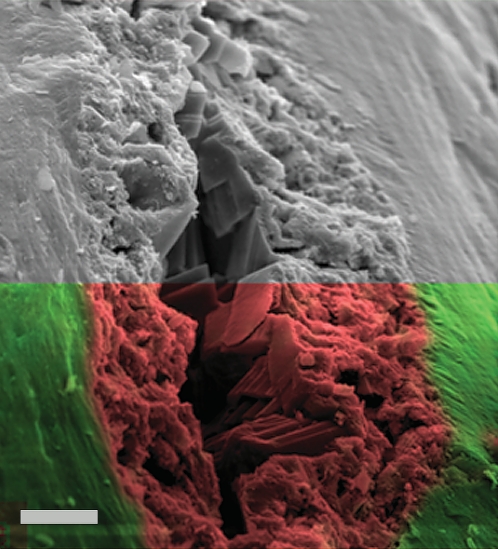
Iron mineralization in vascular canal. SEM image of fractured bone surface across canal. Bottom half is EDS overlay with red representing a mineralized iron coating and green, calcium from the original bone. The transition from bone to coating is not immediately apparent without elemental analysis. UWBM 89326 Scale bar, 10 µm.

The arrows in Fig. 5 identify coatings that peeled away from the bone when fractured, revealing a layered structure. Figure 6 shows an SEM image of a typical vascular canal in the upper half of the image. The bottom is overlaid with an elemental map showing the presence of iron (shown in red) within the coating of the vascular canal, and the presence of calcium (shown in green) in the bone itself. This picture illustrates the difficulty of identifying the layering in a standard image and may have contributed to these coatings not being identified in the past. All of the Lance and Hell Creek specimens collected from both the surface and several meters deep in quarries, showed some evidence of this coating. Most were iron-infiltrated but some contained only carbon and were not mineralized.

**Figure 7:**
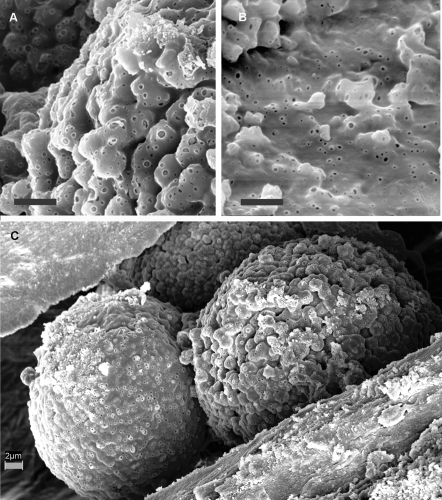
Bubble structures. (A) Bubble-like structures are found throughout some vascular canals. (B) Similar bubble-like structures found on framboids demonstrating that the coatings are undifferentiated between framboids, crystals and canal walls. (C) Framboid on left shows a heavy coating of biofilm that completely obscures the framboidal structure that is still evident on the right specimen UWBM 89322, UWBM 89328 Scale bars, 2 µm.

Many framboids had a surface showing uncharacteristic bubble-like pores (Fig. 7A). These same pores are found on the surface coating of the vascular canal (Fig. 7B). These pores are inconsistent with a mineral origin and show that the same coatings cover the vascular canals, framboids and crystals found in the voids. The coatings on these additional structures imply that this phenomenon is not unique to preserved blood vessels, which would only be present on the walls.

**Figure 8:**
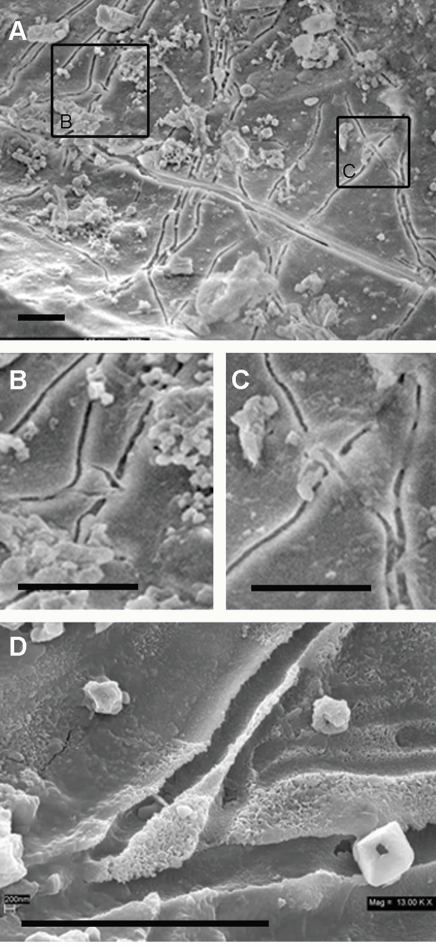
Bridging trough structures. (A) Vascular canal showing crack-like morphologies which are actually troughs, suggesting that organisms moved through a viscous medium. (B and C) Close-ups of bridged structures that are inconsistent with inorganic processes. (D) High magnification of additional trough structures showing rounded bottoms and branching morphology. UWBM 89322 Scale bars, 5 µm.

Figure 8A shows the concave surface of a vascular canal in trabecular bone. A network of structures, originally overlooked in this survey as cracks, cover the surface. The “cracks”, upon closer inspection, bridge each other in a way that is inconsistent with any inorganic process (Fig. 8, B and C). Closer investigation of these structures reveals a trough rather than a crack (Fig. 8D). This data suggests that these “cracks” are formed by free-swimming microbes or bacteria [5] in a viscous medium–again reinforcing the biofilm hypothesis.

**Figure 9:**
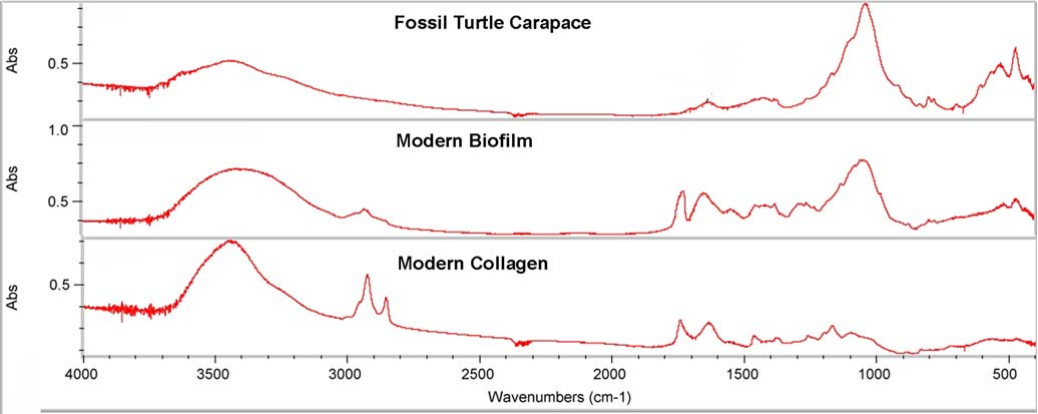
Infrared spectral comparison. Infrared spectra showing similarity of modern biofilms and modern collagen compared to fossil coatings. Cross correlation shows that the fossil material more closely resembles the modern biofilm than the modern collagen.

An experiment was conducted to compare infrared spectra of modern biofilms with modern collagen and fossil bone coatings. Modern biofilms were grown on microscope slides from local pond water with high iron content. These slides developed EDS signatures of iron contamination within 2 weeks of formation. Sample coatings from fossil turtle carapace were submitted to infrared spectroscopy and compared to spectra of modern biofilms and modern collagen (Fig. 9). Fourier cross-correlation showed an 83% match between modern biofilms and fossil specimen with only a 37% correlation to modern collagen. This experiment suggests that modern biofilms share a closer molecular make-up than modern collagen to the coatings from fossil bones.

**Figure 10:**
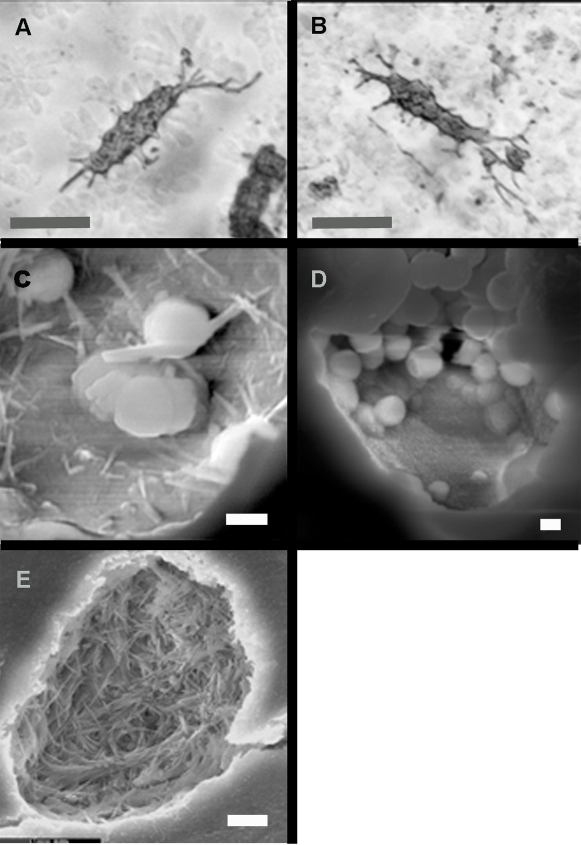
Osteocytes and lacunae. (A and B) Osteocytes found floating free in acid baths with fillapodia. (C,D,E) Fractured lacunae examined with SEM show filaments and spheres consistent with bacterial forms. UWBM 89325, UWBM 89322 Scale bars, A,B 10 µm, C–E 1 µm.

The third structures recovered from the acid baths, were free floating osteocytes complete with fillapodia . (Fig. 10, A and B). Freshly fractured bone shows cross sections though many lacunae. SEM investigation of these lacunae before acid dissolution yielded the following results. Figures 10, C, D and E are several examples of material contained in the original lacunae. The variety of forms found (even in the same bone), indicates that the lacunae are not isolated pockets of exceptional preservation. The structures present are sub-micron spheres and rods, which are morphologically consistent with bacterial structures.

In order to determine if the mineralized biofilms were ancient in origin, a sample of material removed from the vascular canals was subjected to ^14^C dating. The results were ‘greater than modern’ indicating a modern origin for the material.

## Discussion

The iron-oxygen spheres identified here as framboids, ranged in size from 5–29 microns and fall in the range previously described by Schweitzer and Horner [6] with an average size of 25 microns. The elemental signature of these objects differed from the standard pyrite framboid, which includes iron and sulfur. The lack of sulfur was previously used as an argument that these structures were not framboids [6]. The literature shows that pyrite framboids containing sulfur can be oxidized and replaced by iron (hydroxy) oxides over time, leading to a complete loss of sulfur but maintaining the original framboidal structure [7], [8] including those found in dinosaur bones [9]. Biofilms coating these structures can at times obscure the crystal faceting of the original framboid structure (Fig 7,C) further hampering identification. The presence of biofilm coatings on already oxidized framboids suggest their later formation.

This investigation contends that iron-oxygen spheres are far too common in many formations to be the result of extraordinary preservation. Framboid morphology and elemental signature may superficially make them appear to be related to biological structures but they are, in fact, an inorganically produced mineral feature [10] often found in association with organic matter [4]. This idea was originally proposed by Martill and Unwin [11].

The bubble structures suggest the release of gasses from a viscous medium [12]. Various forms of anaerobic bacteria release gaseous by-products as bubbles which erupt on the surface [13]. This suggests that the medium is a desiccated exopolysaccharide glycocalyx known commonly as bacterial biofilm. A biofilm would coat the voids of vascular canals and lacunae, producing an *endocast* of the structure. Once the bone is dissolved, these biofilm endocasts would closely mimic pliable vascular structures. The results presented here suggest that the tubular structures and osteocytes are formed by this process. The lack of observed cell structure in the transparent tubes is inconsistent with preserved tissues.

Recent protein work by Asara et al. [14] examined ground tyrannosaur bone under a highly sensitive mass spectrometer. This resulted in seven recovered protein sequences attributed to the original tyrannosaur but only in femptogram quantities (10^−15^ gram moles). The additional detection of bacterial proteins, identified at the species level as the decomposing bacteria *Rhodococcus* sp. [14] showed conclusively that bacterial contamination was present, even though the original bone was deeply buried, [15]. *Rhodococcus* sp. exhibits morphological differentiation and can be found as both cocci and filaments [16] consistent with forms found in lacunae from this survey (Fig. 10). Recent discoveries of collagen-like proteins in bacteria and viruses [17] add to the problem of unambiguous identification of vertebrate biomolecules.

Biofilms are complex systems produced by virtually all bacteria on almost any water/surface boundary and are ubiquitous in nature [18], [19]. They provide a protective medium against changes in the broader environment from pH levels, toxins, etc. They are viscous, flexible and long lasting through mineralization. Recent biofilms would be naturally pliable and elastic while duplicating the shape of the surfaces they form on. Biofilms harbor ionic bonds which make them pre-disposed to mineralization [20] and is exemplified by calculus on human teeth. Examination of modern biofilms showed copious quantities of bacteria living in the films, however, SEM images only show a smooth undulating profile of the biofilm surface consistent with previous studies [21]. The voids in dinosaur bone provide the micro-environmental equivalent of a natural cave where the discovery of biofilms has become an area of active study.

The detection of similar structures by the previous body of work across time and taxa, suggests an overlap with this survey [2]. When biofilms coat a substrate, and that substrate is subsequently removed, the biofilm will retain much of the original morphology. This can explain the quantity and similarity of structures found in fossil bone and indicates that these structures are unlikely to be preserved dinosaurian tissues but the product of common bacterial activities.
